# Time of Day-Dependent Alterations in Hippocampal Kynurenic Acid, Glutamate, and GABA in Adult Rats Exposed to Elevated Kynurenic Acid During Neurodevelopment

**DOI:** 10.3389/fpsyt.2021.734984

**Published:** 2021-09-17

**Authors:** Courtney J. Wright, Katherine M. Rentschler, Nathan T. J. Wagner, Ashley M. Lewis, Sarah Beggiato, Ana Pocivavsek

**Affiliations:** ^1^Department of Pharmacology, Physiology, and Neuroscience, University of South Carolina School of Medicine, Columbia, SC, United States; ^2^Department of Medical, Oral and Biotechnological Sciences, University of Chieti-Pescara, Chieti, Italy

**Keywords:** kynurenine, NMDA receptor, schizophrenia, psychotic disorders, prenatal

## Abstract

Hypofunction of glutamatergic signaling is causally linked to neurodevelopmental disorders, including psychotic disorders like schizophrenia and bipolar disorder. Kynurenic acid (KYNA) has been found to be elevated in postmortem brain tissue and cerebrospinal fluid of patients with psychotic illnesses and may be involved in the hypoglutamatergia and cognitive dysfunction experienced by these patients. As insults during the prenatal period are hypothesized to be linked to the pathophysiology of psychotic disorders, we presently utilized the embryonic kynurenine (EKyn) paradigm to induce a prenatal hit. Pregnant Wistar dams were fed chow laced with kynurenine to stimulate fetal brain KYNA elevation from embryonic day 15 to embryonic day 22. Control dams (ECon) were fed unlaced chow. Plasma and hippocampal tissue from young adult (postnatal day 56) ECon and EKyn male and female offspring were collected at the beginning of the light (Zeitgeber time, ZT 0) and dark (ZT 12) phases to assess kynurenine pathway metabolites. Hippocampal tissue was also collected at ZT 6 and ZT 18. In separate animals, *in vivo* microdialysis was conducted in the dorsal hippocampus to assess extracellular KYNA, glutamate, and γ-aminobutyric acid (GABA). Biochemical analyses revealed no changes in peripheral metabolites, yet hippocampal tissue KYNA levels were significantly impacted by EKyn treatment, and increased in male EKyn offspring at ZT 6. Interestingly, extracellular hippocampal KYNA levels were only elevated in male EKyn offspring during the light phase. Decreases in extracellular glutamate levels were found in the dorsal hippocampus of EKyn male and female offspring, while decreased GABA levels were present only in males during the dark phase. The current findings suggest that the EKyn paradigm may be a useful tool for investigation of sex- and time-dependent changes in hippocampal neuromodulation elicited by prenatal KYNA elevation, which may influence behavioral phenotypes and have translational relevance to psychotic disorders.

## Introduction

Disruptions in neurotransmission are associated with the pathology of psychotic disorders such as schizophrenia (SZ) and bipolar disorder (BD). In particular, dysregulated modulation of the excitatory neurotransmitter glutamate and the inhibitory small molecule y-aminobutyric acid (GABA) has been implicated in the etiology of cognitive, negative, and positive symptoms in individuals with severe psychiatric illness (1–4). Hypofunction of the cortical ionotropic glutamate receptor N-methyl-d-aspartate (NMDA) is thought to contribute to dysregulated tonic GABAergic inhibition, alterations in cortical glutamate levels, and the pathophysiological manifestation of cognitive and negative symptoms in individuals with SZ (2).

**Figure 1:**
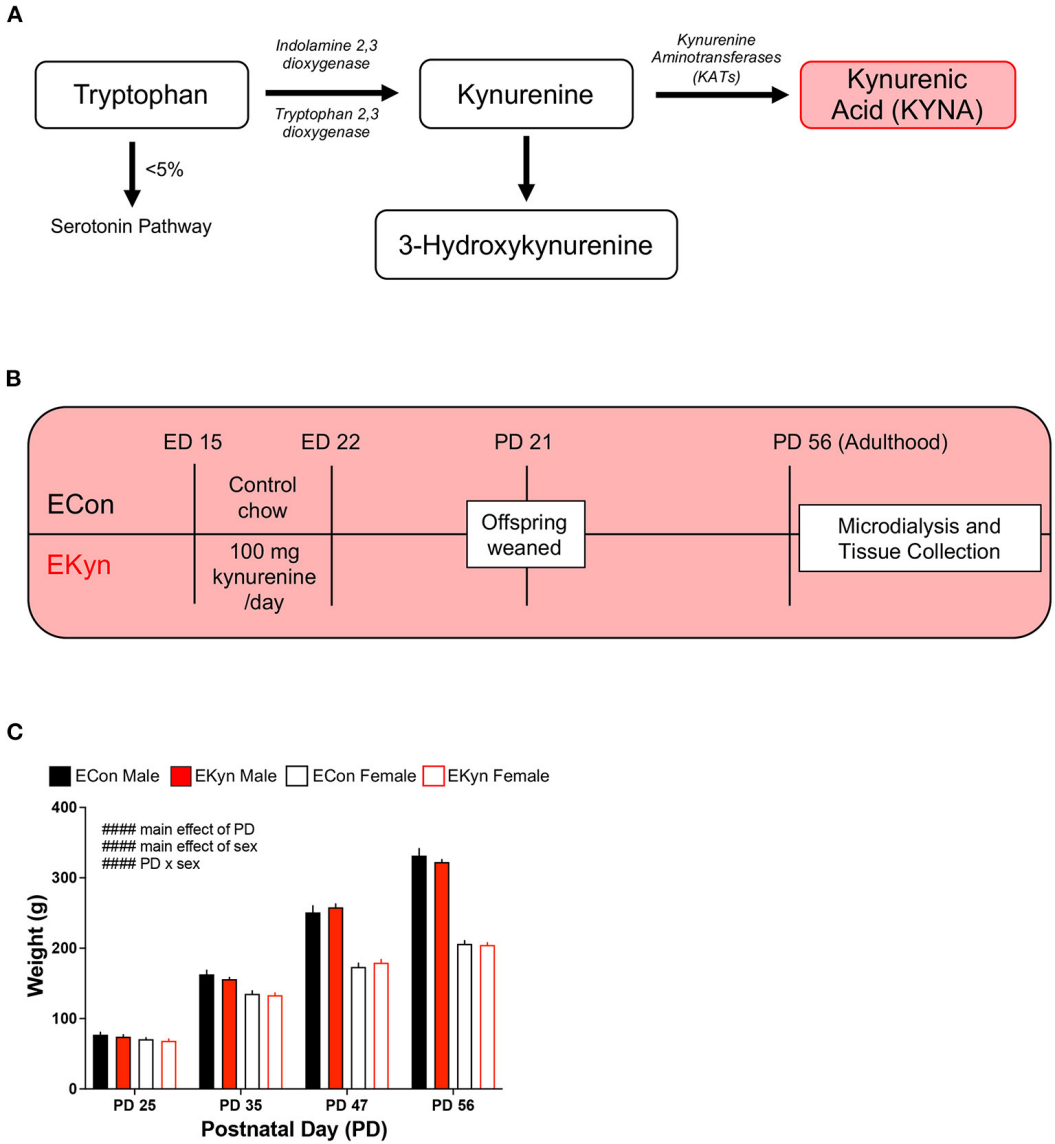
Schematic showing the kynurenine pathway, experimental paradigm, and rat body weight across age. **(A)** A simplified schematic of the kynurenine pathway of tryptophan degradation, wherein kynurenic acid (KYNA) is synthesized from kynurenine via kynurenine aminotransferases (KATs). **(B)** EKyn experimental paradigm: pregnant rat dams are fed normal rodent chow (ECon) or rodent chow laced with 100 mg of kynurenine (EKyn) daily from embryonic day (ED) 15 to ED 22. Male and female offspring are weaned at postnatal day (PD) 21 and used in experiments at PD 56, when they reach adulthood. **(C)** Body weight of ECon and EKyn offspring at PD 25, PD 35, PD 47, and PD 56. Data are mean ± SEM. Repeated measures 3-way ANOVA effects (#### *P* < 0.0001) followed by Bonferroni's *post-hoc* test. *n* = 4–8 litters per group.

Abnormally high levels of the endogenous neuromodulator and tryptophan metabolite kynurenic acid (KYNA) (Figure 1A) are found in the brain and cerebrospinal fluid of individuals with SZ and BD (5–10). KYNA is of particular interest as it competitively antagonizes NMDA receptors at the glycine site, and inhibits α7 nicotinic acetylcholine (α7nACh) receptors, thereby directly influencing neurotransmission (11–14). Elevated KYNA is hypothesized to be causally related to neurocognitive impairments in patients with psychotic disorders (15). Preclinical studies in animal models postulate that increased KYNA impairs learning and memory, especially in brain regions like the prefrontal cortex and hippocampus, whereas KYNA reductions may feasibly improve learning and memory (16–22).

SZ and BD are classified as neurodevelopmental disorders, and perinatal insults, such as stress or infection, associated with these diseases can result in the activation of the kynurenine pathway (KP) and increase levels of KYNA. Further, the prenatal period has been found critical for elevations in KYNA to cause long term biochemical changes and cognitive dysfunction in adult rats (23–26). Hence, to further investigate the neurodevelopmental impacts of KYNA elevation, we utilize the embryonic kynurenine (EKyn) paradigm in rats, wherein pregnant Wistar dams are fed 100 mg of kynurenine-laced chow daily from embryonic day (ED) 15 to ED 22 (25, 27) (Figure 1B). This time course corresponds to the second trimester in human pregnancy, when the developing fetus is most vulnerable to exposure from infection or injury, thereby providing a translational model for *in utero* insults that instigate neurodevelopmental abnormalities (28–30). Substantial evidence also suggests that rodents subjected to elevated KYNA during this critical prenatal window will exhibit long-lasting deficits in adulthood (25, 31–35).

We recently determined conspicuous sex and time of day dependent changes in sleep, home cage activity, and arousal in young adult EKyn offspring (27). In a behavioral context, sleep and arousal states depend on hippocampal neuromodulation to regulate memory consolidation, retrieval, and locomotor activity (36, 37). Thus, our present aim was to investigate underlying abnormalities in levels of excitatory neurotransmitter glutamate and inhibitory neurotransmitter GABA, in relation to KYNA, in the hippocampus of young adult EKyn offspring. We hypothesized sex- and time-dependent changes in hippocampal GABAergic and glutamatergic neurotransmission in adulthood as a result of prenatal KYNA elevation. Of translational relevance, kynurenine pathway metabolites are modulated in a circadian-dependent manner in humans, with excreted metabolite levels peaking mid-morning after tryptophan administration (38). Therefore, we also evaluated KP metabolites in the plasma of young adult EKyn rats. Importantly we determined that while central levels of KYNA and neurotransmitters change in time of day and sex-dependent manners in our EKyn paradigm, plasma metabolites do not serve as predictors for changes in the brain. Interestingly, while KYNA levels were elevated in EKyn males, extracellular glutamate levels were attenuated in both EKyn males and females, yet GABA attenuation was only evident in EKyn males. Our study highlights sex differences in response to prenatal KYNA elevation and its impact on hippocampal neuromodulation of GABA and glutamate through altered cerebral KP metabolism.

## Methods

### Animals

Pregnant, adult Wistar rats (ED 2) were obtained from Charles River Laboratories, acclimated to our animal facility, and fed laced diet (details below) beginning on ED 15. All animals were kept on a 12/12 h light-dark cycle, where Zeitgeber time (ZT) 0 corresponded to lights on and ZT 12 corresponded to lights off. The animal facility at the University of South Carolina School of Medicine is accredited by the American Association for the Accreditation of Laboratory Animal Care. All protocols were approved by the University of South Carolina Institutional Animal Care and Use Committees and were in accordance with the National Institutes of Health *Guide for the Care and Use of Laboratory Animals* (39).

### EKyn Treatment

Beginning on ED 15, pregnant dams are fed a wet mash of ground control chow (ECon) or a mash of chow laced with 100 mg of kynurenine (EKyn) daily until ED 22, as previously described (25). Upon birth, dams received normal rodent chow pellets *ad libitum*. On postnatal day (PD) 21, offspring were weaned and pair-housed by sex. The offspring were weighed at PD 25, PD 35, PD 47, and PD 56, but otherwise remained experimentally undisturbed until they reached young adulthood at PD 56 (Figure 1B). A maximum of two rats per sex from a single prenatal litter were used within each experimental cohort to obtain a minimum *n* = 4 litters per experiment.

### Chemicals

L-Kynurenine sulfate salt (“kynurenine,” purity: 99.4%) was obtained from Sai Advantium (Hyderabad, India). All other chemicals were obtained from various suppliers but were of the highest commercially available purity.

### Tissue Collection

Cohorts of offspring were euthanized via CO_2_ asphyxiation at ZT 0, ZT 6, ZT 12, or ZT 18 to collect tissue. Whole trunk blood was collected into tubes containing K_3_-EDTA (0.15%) and centrifuged at 300 × g for 10 min to separate plasma. Brains were promptly removed, and the hippocampus was dissected. All samples were snap frozen on dry ice and stored at −80°C until biochemical analyses.

### Microdialysis

#### Surgery

Under isoflurane anesthesia (2–5%), animals were placed on a stereotaxic frame (Stoelting Co., Wood Dale, IL, USA). Carprofen was used as an analgesic and given at a dose of 5 mg/kg (subcutaneous) at the beginning of surgery. A guide cannula (1.0 mm outer diameter; SciPro Inc., Sanborn, NY, USA) was positioned over the dorsal hippocampus (AP: −3.4, LM: ± 2.3, DV: −1.5 from bregma after coordinates) and anchored in place using two surgical screws inserted into 0.5 mm burr holes and acrylic dental cement. After 24–48 h of post-operative recovery, microdialysis experiments were initiated in freely moving animals.

#### Extracellular Fluid Collection by *in vivo* Microdialysis

Special attention was given to time of day of microdialysis experiments and experimental efforts were made to collect microdialysate for up to 24 h. To control for the contribution of the experimental start time, cohorts of animals were initiated with microdialysis perfusion at ZT 3, ZT 6, ZT 9, or ZT 22.5. On the day of microdialysis, a probe (2 mm PES membrane/14 mm shaft, 6 kD; SciPro Inc.) was inserted through the guide cannula in freely moving animals and a microperfusion pump (Harvard Apparatus, Holliston, MA, USA) set to a flow rate of 2.5 μL/min perfused Ringer solution (147 mM NaCl, 4 mM KCl, 1.4 mM CaCl_2_) through the probe inlet. After 30 min, the flow was reduced to 1.0 μL/min for the duration of the experiment. Collection of dialysate samples began 2 h after the onset of perfusion for KYNA analysis. Glutamate and GABA were analyzed in dialysate samples collected at 4 h after the onset of perfusion, to achieve stable neurotransmitter levels (40). Extracellular KYNA, glutamate, and GABA were analyzed from the same hour fractions and analysis of data was divided by light phase fractions (ZT 0 – ZT 12) and dark phase fractions (ZT 12 – ZT 24). Samples were stored at −80°C until biochemical analyses.

At the end of the experiment, the probe was removed, and each animal was anesthetized using isoflurane, decapitated via guillotine, and the brain was carefully removed and dropped in a 10% formalin solution. Brains were moved step-wise to 20% sucrose before processing with 25–30 μm thick coronal cryostat section that were stained in neutral red to check proper microdialysis cannula placement (Supplementary Figure 1).

### Biochemical Analysis

#### Plasma and Brain (Tryptophan, Kynurenine, KYNA)

On the day of biochemical analyses, plasma samples were thawed, diluted (1:1000 for tryptophan, 1:10 for kynurenine and KYNA), acidified with 6% perchloric acid, and centrifuged at 12,000 × g for 10 min. The hippocampus was weighed, diluted 1:5 (w/v) with ultrapure water, and homogenized with a sonicator. Protein was evaluated in the stock homogenate using the previously published Lowry method (41). A portion of the remaining hippocampal homogenate was further diluted with ultrapure water to a final concentration of 1:10, acidified using 25% perchloric acid, and centrifuged at 12,000 × g for 10 min.

Acidified plasma samples were evaluated for tryptophan, kynurenine, and KYNA and hippocampal samples were evaluated for KYNA by high-performance liquid chromatography (HPLC) analysis as previously described (26). Briefly, 20 μL of supernatant was injected into a ReproSil-Pur C18 column (4 × 150 mm; Dr. Maisch Gmbh, Ammerbuch, Germany) using a mobile phase of 50 mM sodium acetate, pH adjusted to 6.2 with glacial acetic acid, and 5% acetonitrile at a flow rate of 0.5 mL/min. A post column addition of 500 mM zinc acetate at a flow rate of 0.1 mL/min was used to fluorometrically detect tryptophan [excitation (ex): 285, emission (em): 365, retention time (rt): 11 min], kynurenine (ex: 365, em: 480, rt: 6 min), and KYNA (ex: 344, em: 398, rt: 11 min) in the eluate (Alliance, 2,475 fluorescence detector; Waters, Bedford, MA, USA). Data was analyzed using Empower 3 software (Waters).

#### Microdialysate (KYNA)

Extracellular KYNA was assessed by diluting the microdialysate sample 1:2 in ultrapure water and subjecting to fluorometric HPLC, as described above. Microdialysis data were not corrected for recovery from dialysis probe.

#### Microdialysate (Glutamate/GABA)

Extracellular glutamate and GABA from microdialysis samples were assessed using electrochemical ultra-high-performance liquid chromatography (UHPLC) ALEXYS analyzer with a Decade Elite detector (Antec Scientific, Zoeterwoude, Netherlands). Briefly, 9 μL of undiluted microdialysate was injected into a HSS T3 column (1.0 × 50 mm; Waters) using a step gradient elution comprised of the first mobile phase (base solution: 50 mM phosphoric acid, 50 mM citric acid, and 0.1 mM EDTA at a pH of 3.5) and 2% acetonitrile followed by the second mobile phase made from base solution and 50% acetonitrile. Each mobile phase is delivered at a flow rate of 200 μL/min. An in-needle derivatization added 5 μL of *o-*phthaldialdehyde reagent before eluting through the column. A VT03 microflow cell with a 0.7 mm glassy carbon working electrode was used for electrochemical detection (42). Data was acquired using Clarity 8 software (DataApex, Prague, Czech Republic). Microdialysis data were not corrected for recovery from dialysis probe.

### Statistical Analysis

All statistical analyses were performed using Prism 9.0 (GraphPad Software, San Diego, CA, USA), and all results and samples sizes are shown in statistical tables (Supplementary Materials). Weight data were averaged across litters and assessed by 3-way repeated measures ANOVA with EKyn treatment, age, and sex as between-subject factors. Separate analyses by sex were performed by 2-way repeated measures ANOVA with EKyn treatment and age as between-subject factors. From weight data, Bonferroni's *post hoc* test was used for multiple comparisons. Plasma and brain metabolite data were averaged across litters and assessed by 3-way ANOVA with EKyn treatment, sex, and ZT as between-subject factors. Separate analyses by sex were performed by 2-way ANOVA with EKyn treatment and ZT as between-subject factors. Microdialysis data were averaged across litter depending on the start time of the experiment, with groups divided by early-light (ZT 3), mid-light (ZT 6), late-light (ZT 9), and late-dark (ZT 22.5). Samples below the limit of detection for individual analytes were not included in those respective analyses. Microdialysis data were analyzed separately by phase by 3-way ANOVA with EKyn treatment, sex, and ZT as between-subject factors. Separate analysis by sex was performed in each phase by 2-way ANOVA with EKyn treatment and ZT as between-subject factors. Analyses were followed up by appropriate 2-way interactions. Uncorrected Fisher's LSD was used for multiple comparisons in analysis of biochemical data. Statistical significance was defined as *P* < 0.05.

## Results

### Sex, but Not Prenatal KYNA Elevation, Influences the Weight of EKyn and ECon Offspring

To determine if elevated prenatal KYNA exposure impacts the body weight of offspring during adolescence and young adulthood, we weighed EKyn and ECon offspring at PD 25, PD 35, PD 47, and PD 56. We determined main effects of postnatal day (*F*_3,48_ = 795.8, *P* < 0.0001) and sex (*F*_1,48_ = 1906, *P* < 0.0001) and a significant postnatal day x sex interaction (*F*_3,48_ = 474.7, *P* < 0.0001) (Figure 1C). The body weight of males was consistently greater than females from PD 35, and this difference steadily increased across postnatal development. Of importance, body weight was not impacted by prenatal KYNA elevation in male or female offspring, complementing what has been previously described only in males (34).

### Hippocampal KYNA Levels, but Not Peripheral KP Metabolites, Are Elevated in Young Adult EKyn Offspring

**Figure 2:**
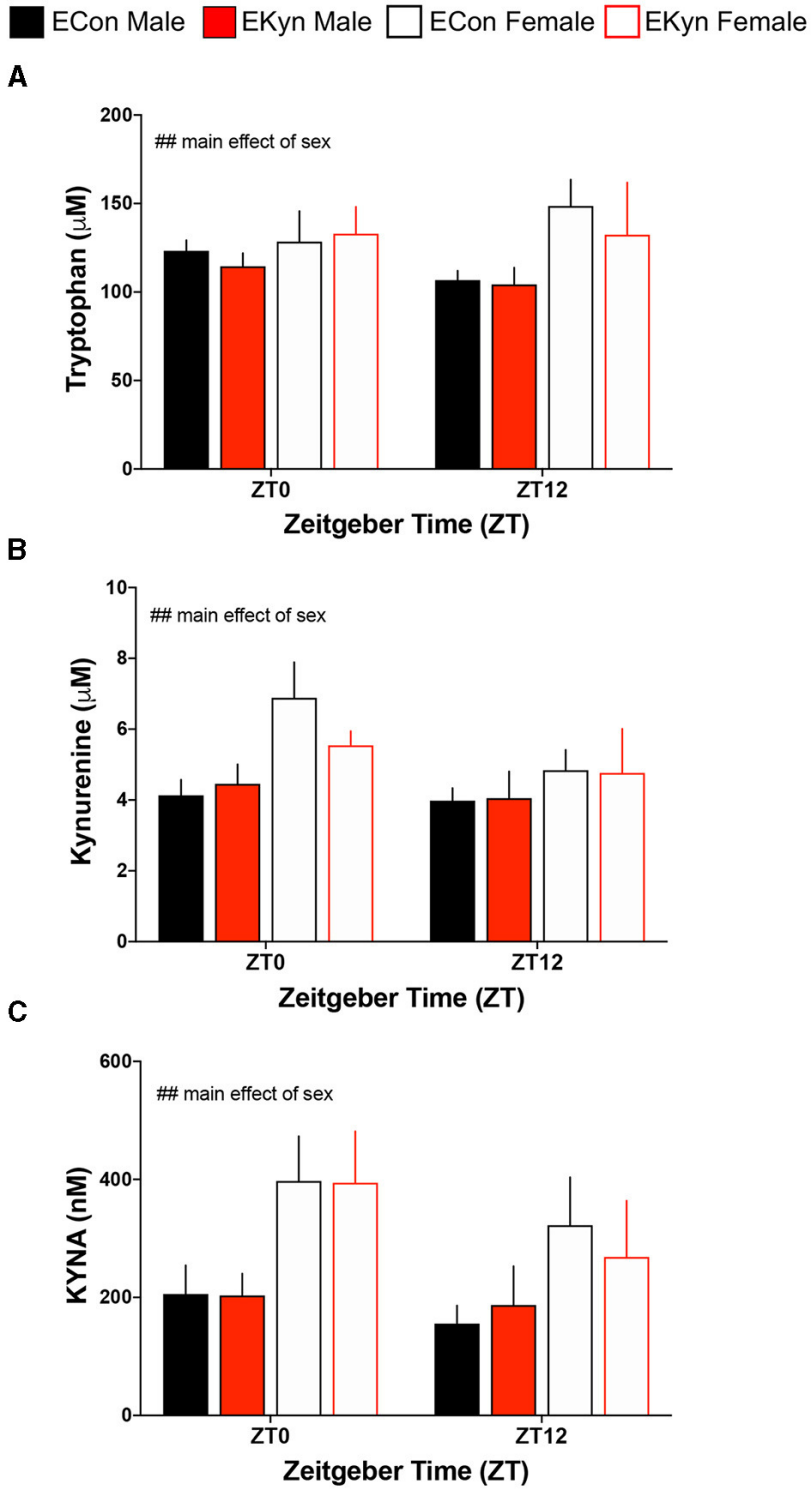
Prenatal KYNA elevation elicits no changes in peripheral kynurenine pathway metabolism in young adult male EKyn. **(A)** Plasma tryptophan. **(B)** Plasma kynurenine. **(C)** Plasma KYNA. All data are mean ± SEM. Three-way ANOVA analyses effects: ## *P* < 0.01. *n* = 3–9 litters per group.

**Figure 3:**
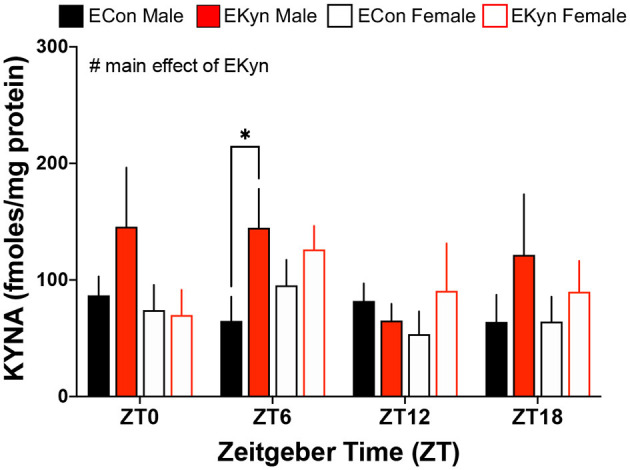
Prenatal KYNA increases hippocampal KYNA in young adult male EKyn offspring. Hippocampal KYNA. All data are mean ± SEM. Three-way ANOVA analyses effects: # *P* < 0.05. 2-way ANOVA analyses by sex followed by Fisher's LSD *post-hoc* test: *P < 0.05. *n* = 4–12 litters per group.

To evaluate circadian dynamics of KP metabolism, we first measured peripheral and hippocampal KP metabolites at specific time points during the light and dark phases. Plasma tryptophan (Figure 2A), kynurenine (Figure 2B), and KYNA (Figure 2C) were not impacted by EKyn treatment at the beginning of the light phase, ZT 0, or at the beginning of the dark phase, ZT 12. Peripheral metabolites tryptophan (*F*_1,40_ = 7.658, *P* = 0.0085), kynurenine (*F*_1,41_ = 7.640, *P* = 0.0085), and KYNA (*F*_1,41_ = 11.53, *P* = 0.0015) were significantly impacted by sex, as we determined that females had elevated metabolites compared to males. Hippocampal KYNA was significantly impacted by EKyn treatment (*F*_1,107_ = 4.879, *P* = 0.0293), with increased KYNA in hippocampal tissue in EKyn across the light phase, and *post-hoc* in EKyn males at ZT6 compared to ECon (*P* = 0.0500; Figure 3).

### Prenatal KYNA Elevation Elicits an Increase in Extracellular KYNA Levels During the Light Phase in the Dorsal Hippocampus of Young Adult EKyn Males

**Figure 4:**
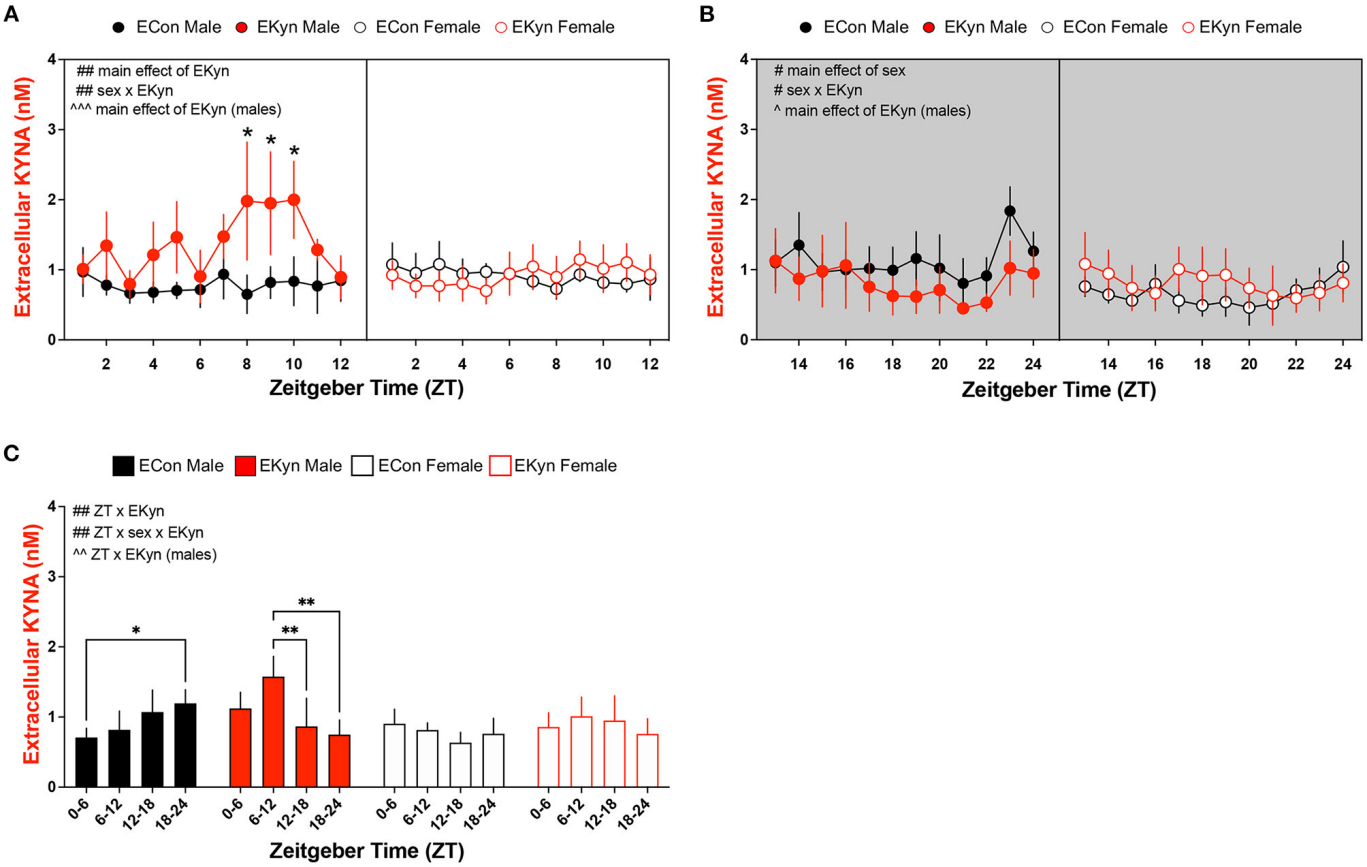
Extracellular KYNA in the hippocampus is increased during the light phase and decreased during the dark phase in EKyn male offspring. Microdialysis in the dorsal hippocampus was conducted in young adult offspring, with special attention given to the time of day. Data are represented by phase, wherein light phase denotes ZT 0–12 and dark phase denotes ZT 12–24. **(A)** Light phase. **(B)** Dark phase. **(C)** Analysis of 6-h bins across light and dark phase. Data are mean ± SEM. 3-way ANOVA analyses effects: # *P* < 0.05, ## *P* < 0.01. 2-way ANOVA analyses by sex effects: ^∧^*P* < 0.05, ^∧∧^*P* < 0.01, ^∧∧∧^*P* < 0.001. Fishers LSD *post-hoc* test: *P < 0.05, **P < 0.01. *n* = 4–8 litters per group.

To more precisely investigate circadian-dependent alterations in KYNA levels, we analyzed extracellular KYNA in the dorsal hippocampus of EKyn and ECon young adult offspring. During the light phase, extracellular KYNA was impacted by a main effect of EKyn treatment (*F*_1,207_ = 10.62, *P* = 0.0013 and a sex x EKyn treatment interaction (*F*_1,207_ = 10.01, *P* = 0.0018) (Figure 4A). In males, extracellular KYNA was significantly influenced by EKyn treatment (*F*_1,87_ = 13.39, *P* = 0.0004), and EKyn males experienced elevated extracellular KYNA in the latter half of the light phase (ZT 8, *P* = 0.0282; ZT 9, *P* = 0.0312; ZT 10, *P* = 0.0367). Extracellular KYNA in female EKyn offspring, however, remained unchanged compared to female ECon offspring in the light phase. Within the dark phase, extracellular KYNA was significantly impacted by a main effect of sex (*F*_1,160_ = 6.635, *P* = 0.0109) and a sex x EKyn treatment interaction (*F*_1,160_ = 6.744, *P* = 0.0103) (Figure 4B). In males, extracellular KYNA was reduced in the EKyn group (*F*_1,68_ = 4.556, *P* = 0.0364), but not altered in EKyn females compared to controls. We also analyzed averaged 6-h bins of microdialysis data to evaluate the contribution of early light phase (ZT 0–6), late light phase (ZT 6–12), early dark phase (ZT 12–18) or late dark phase (ZT 18–24) on extracellular KYNA levels. We determined that extracellular KYNA was impacted by a significant ZT x EKyn treatment interaction (*F*_3,46_ = 6.364, *P* = 0.0011) and a three-way ZT x sex x EKyn treatment interaction (*F*_3,46_ = 5.242, *P* = 0.0034) (Figure 4C). When analyses were separated by sex, we determined in males that extracellular KYNA was impacted by a ZT x EKyn treatment interaction (*F*_3,19_ = 5.279, *P* = 0.0081). In ECon males, extracellular KYNA was elevated at the end of the dark phase when compared to the light phase (ZT 18–24 vs. ZT 0–6, *P* = 0.0440), while in EKyn males extracellular KYNA was reduced across the entire dark phase when compared to the light phase (ZT 12–18 vs. ZT 6–12, *P* = 0.0091; ZT 18–24 vs. ZT 6–12, *P* = 0.0038). In females, extracellular KYNA was not influenced by EKyn treatment or time of day.

### Reduced Extracellular Glutamate in Young Adult EKyn Offspring

**Figure 5:**
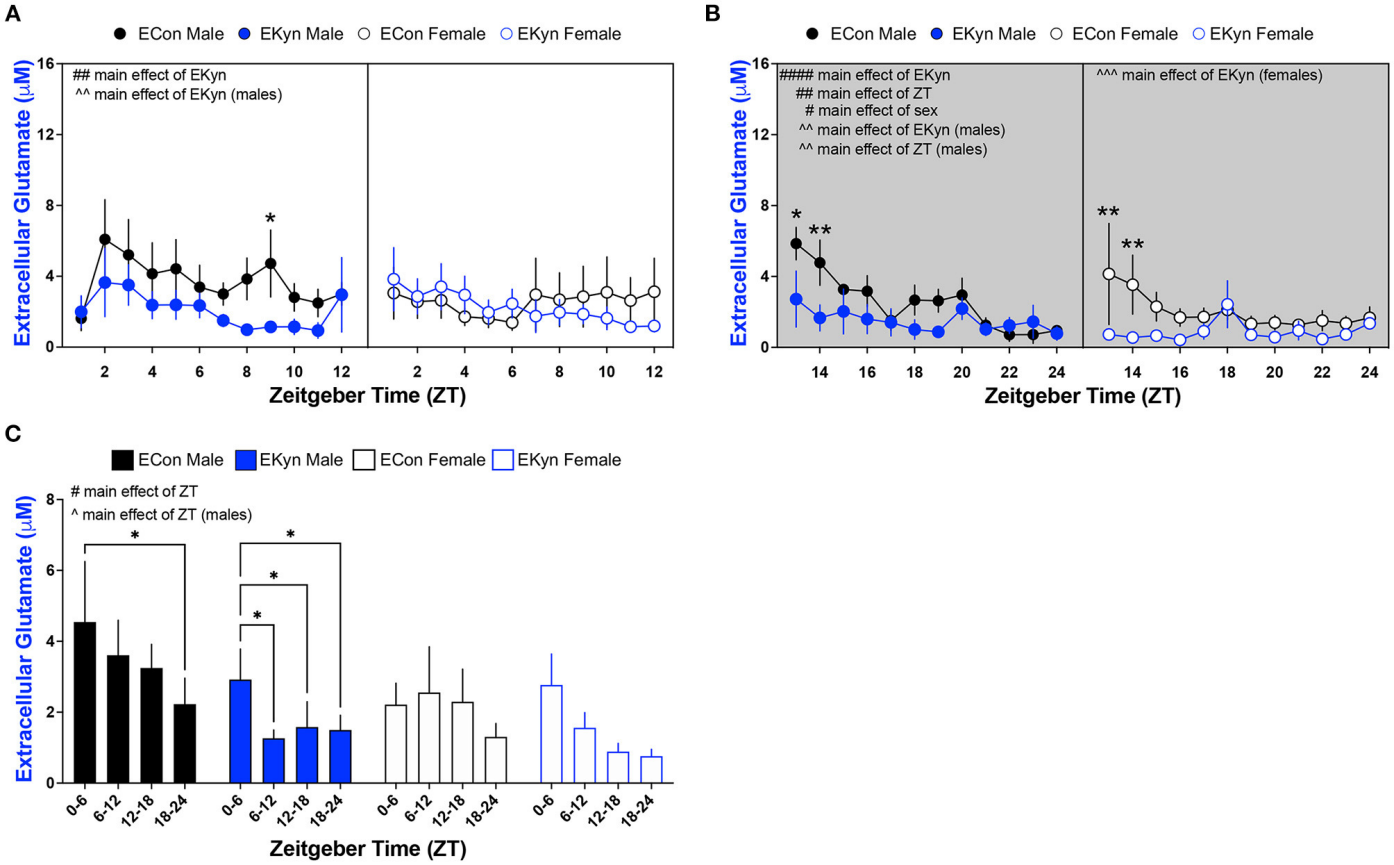
Light phase- and sex-dependent alterations in extracellular glutamate in the hippocampus of young adult offspring exposed to elevated prenatal KYNA. Microdialysis in the dorsal hippocampus was conducted in young adult offspring, with special attention given to the time of day. Data are represented by phase, wherein light phase denotes ZT 0–12 and dark phase denotes ZT 12–24. **(A)** Light phase. **(B)** Dark phase. **(C)** Analysis of 6 - h bins across light and dark phase. Data are mean ± SEM. 3-way ANOVA analyses effects: # *P* < 0.05, ## *P* < 0.05, #### *P* < 0.0001. 2-way ANOVA analyses by sex effects: ^∧^*P* < 0.05, ^∧∧^*P* < 0.01. ^∧∧∧^*P* < 0.001. Fishers LSD *post-hoc* test: *P < 0.05, **P < 0.01. *n* = 5–12 litters per group.

To test the hypothesis that elevated KYNA influences neurotransmitter levels, we evaluated levels of extracellular glutamate and GABA in EKyn and ECon offspring in the dorsal hippocampus. EKyn treatment significantly influenced extracellular glutamate during the light phase (*F*_1,311_ = 6.984, *P* = 0.0086) (Figure 5A). EKyn males, in particular, had reduced extracellular glutamate during the light phase when compared to controls (*F*_1,113_ = 8.616, *P* = 0.0040), but this reduction was not present in EKyn females. In the dark phase, extracellular glutamate was significantly impacted by main effects of ZT (*F*_11,179_ = 2.941, *P* = 0.0013), EKyn treatment (*F*_1,179_ = 22.40, *P* < 0.0001), and sex (*F*_1,179_ = 6.416, *P* = 0.0122) (Figure 5B). Glutamate was reduced by the end of the dark phase in male and female ECon and EKyn offspring, and lower in females than in males. Further, we determined that EKyn treatment resulted in reduced extracellular glutamate in both male (*F*_1,71_ = 9,772, *P* = 0.0026) and female (*F*_1,108_ = 13.77, *P* = 0.0003) offspring compared to counterpart ECon in the dark phase. The time of day, ZT, impacted extracellular glutamate levels in EKyn males during the dark phase (*F*_11,71_ = 2.917, *P* = 0.0032). When we evaluated averaged 6-h bins, we determined that the time of day significantly influenced extracellular glutamate (*F*_3,68_ = 4.034, *P* = 0.0106) (Figure 5C). EKyn males sustained reduced glutamate after ZT 6 (ZT 6–12 vs. ZT 0–6, *P* = 0.0303; ZT 12–18 vs. ZT 0–6, *P* = 0.0318; ZT 18–24 vs. ZT 0–6, *P* = 0.0258) and EKyn females after ZT 12 (ZT 12–18 vs. ZT 0–6, *P* = 0.0422; ZT 18–24 vs. ZT 0–6, *P* = 0.0460) when compared to the first 6 h of the light phase.

### Prenatal KYNA Elevation Elicits Sex-Dependent Changes in Extracellular GABA in Young Adult Offspring

**Figure 6:**
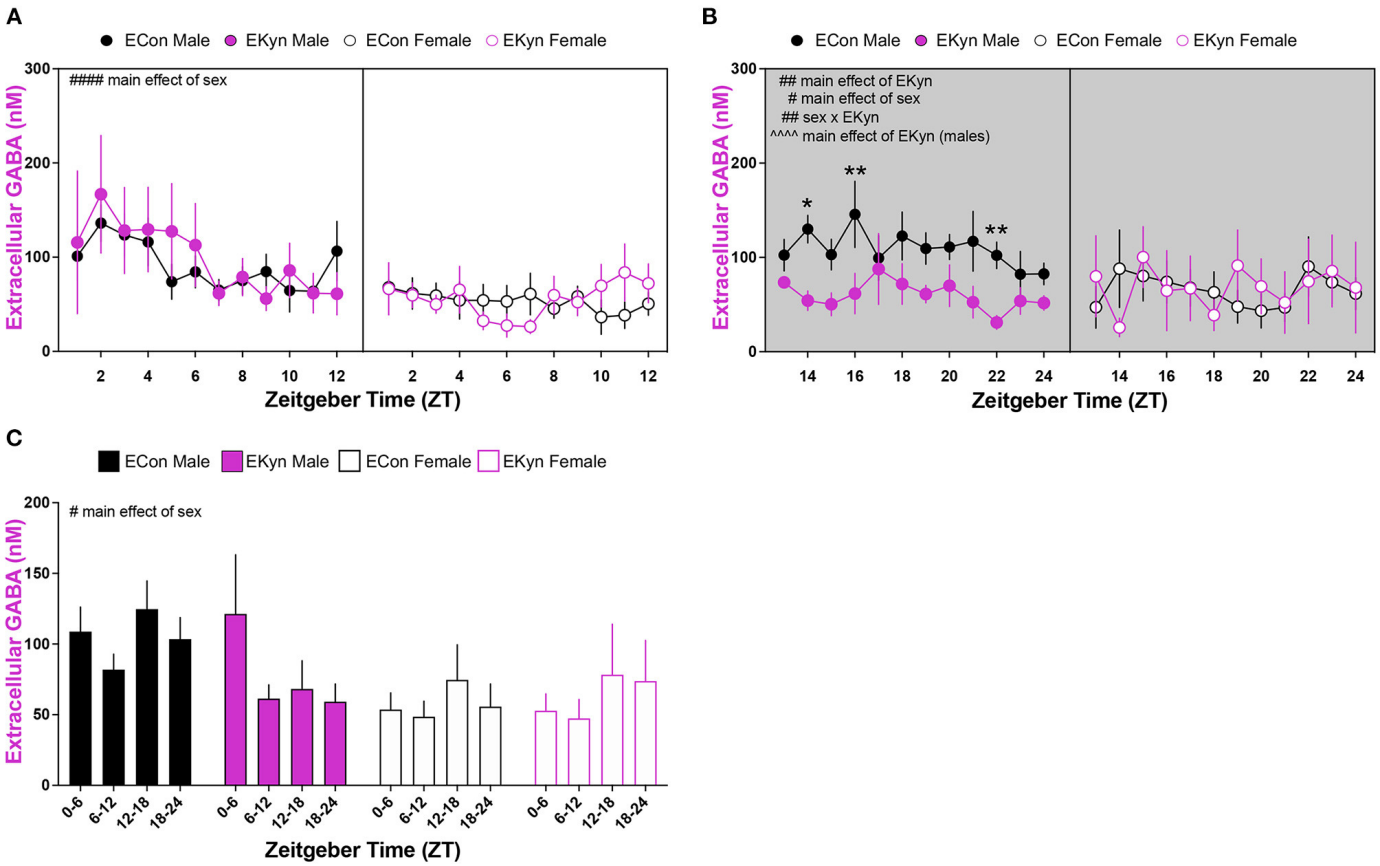
Reduced extracellular GABA levels in the hippocampus during the dark phase in young adult male EKyn. Microdialysis in the dorsal hippocampus was conducted in young adult offspring, with special attention given to the time of day. Data are represented by phase, wherein light phase denotes ZT 0–12 and dark phase denotes ZT 12–24. **(A)** Light phase. **(B)** Dark phase. **(C)** Analysis of 6-hour bins across light and dark phase. Data are mean ± SEM. 3-way ANOVA analyses effects: # *P* < 0.05, ## *P* < 0.01, #### *P* < 0.0001. 2-way ANOVA analyses by sex effects: ^∧∧∧∧^*P* < 0.0001. Uncorrected Fishers LSD *post-hoc* test: *P < 0.05, **P < 0.01. *n* = 4–11 litters per group.

Lastly, we determined conspicuous disturbances in extracellular GABA in the hippocampus of young adult EKyn offspring. In the light phase, extracellular GABA was influenced by a main effect of sex (*F*_1,256_ = 32.54, *P* < 0.0001), but not time of day or EKyn treatment (Figure 6A). However, in the dark phase, we determined significant main effects of EKyn treatment (*F*_11,166_ = 7.170, *P* = 0.0082) and sex (*F*_1,166_ = 4.213, *P* = 0.0417), and a significant sex x EKyn treatment interaction (*F*_1,166_ = 9.017, *P* = 0.0031) (Figure 6B). Male EKyn offspring had reduced extracellular GABA when compared to controls (*F*_1,76_ = 23.00, *P* < 0.0001) in the dark phase. When 6-h bins were evaluated, we determined that extracellular GABA levels were significantly impacted by sex (*F*_1,31_ = 6.548, *P* = 0.0156), such that extracellular GABA was reduced in females compared to males (Figure 6C).

## Discussion

We presently confirmed that prenatal KYNA elevation results in elevated tissue KYNA levels and extracellular KYNA levels in the hippocampus of young adult male EKyn offspring (21, 25, 26). Of interest, our current focus extensively evaluated the contribution of the time of day of experimentation, while also expanding our understanding of biochemical dynamics in both sexes of EKyn offspring. Our results reinforce previous findings that the long term consequences of prenatal KYNA elevation manifest in the attenuation of glutamate levels in the rat hippocampus (21) and complement our recent characterization of sex-dependent diurnal changes in sleep and arousal behaviors in EKyn offspring (27). As no differences in weight were observed between EKyn and control offspring, we presently provide critical evidence, in both sexes, that the reported long-term manifestation of prenatal KYNA elevation are not attributed to body weight differences.

Consistent with our previous evaluation of KP metabolites in the plasma of EKyn offspring (26, 27), plasma tryptophan, kynurenine, and KYNA remained unchanged between experimental groups at ZT 0 and ZT 12. Within the brain however, KYNA levels in dissected hippocampal tissue were significantly elevated in male EKyn offspring during the middle of the light phase (ZT 6), supporting our findings from previous studies evaluating brain tissue KYNA content in EKyn compared to ECon offspring (25–27, 33). We presently selected the time points that correspond to transitions between the light and dark phases for rodents, as we previously studied time points that corresponded to the middle of the light and dark phases for rodents (27). As such, we determined that female offspring had conspicuously higher tryptophan, kynurenine, and KYNA levels in the plasma compared to males. However, levels of KP metabolites in the periphery did not serve as strong predictors for the observed changes in brain KYNA, though perhaps limited by time intervals of plasma sampling in our animals. As brain KP metabolism is uniquely regulated (43), peripheral KP metabolism in clinical studies especially may limit the understanding of changes in the central nervous system (5, 7, 15, 44).

Notably, KYNA in the hippocampus, both tissue content and extracellular levels, were elevated in male EKyn offspring during the light phase, followed by a sustained decrease in levels during the dark phase. KYNA elevation during the light phase corresponds to evolutionarily conserved circadian rhythmicity of tryptophan catabolism in rodents and humans (38, 45). This diurnal pattern of KYNA modulation in EKyn male offspring corresponds with concurrent glutamate attenuation across the light and first 4 h of the dark phase yet sustained normal levels of the neurotransmitter during the latter half of the dark phase. Most notably, when extracellular hippocampal KYNA levels decrease from ZT 18 to ZT 24 in EKyn males, extracellular glutamate stabilizes to levels comparable to ECon males, suggesting that KYNA levels are influencing extracellular glutamate fluctuations. This notion is supported by evidence that acute elevations of KYNA, in a dose-dependent manner, result in locally reduced glutamate levels in several brain regions, including the hippocampus (18, 46), and further reinforced by restoration of glutamate levels when KYNA levels are modulated via kynurenine amino transferase II (KAT II) inhibition or the α7nACh positive allosteric modulator, galantamine (18, 19, 47, 48). Pharmacological intervention with galantamine or a KAT II inhibitor has also been shown to restore cognitive flexibility and glutamate levels in offspring exposed to elevated KYNA during neurodevelopment, further supporting the notion that these neurochemical alterations are related to the neuromodulatory properties of KYNA (19, 21).

In adult female EKyn offspring, extracellular KYNA was not elevated extracellularly. However, glutamate levels were found to be reduced during the first 6 h of the dark phase compared to counterpart controls. It is important to note that the exact relationship between KYNA and its impact on extracellular neurotransmitters in females specifically remains understudied, as most acute, dose-response pharmacological studies have been conducted only in male rodents (18, 40, 46, 49, 50). Attenuated glutamate levels in EKyn offspring could be related to changes in local synaptic connections and dendritic morphology in adult animals exposed to high levels of KYNA during neurodevelopment (23, 24, 33, 34). Conspicuously, the alterations in glutamate presently characterized may shed insight on our recent determination of altered arousal patterns in female EKyn offspring, specifically reduced home cage activity and prolonged bouts of wakefulness during the dark phase (27). Aside from glutamate levels, future studies will be critical to determine if EKyn offspring suffer from an overall reduction of neurotransmission which may thereby influence the array of neurocognitive impairments determined in these animals (21, 25, 26, 34, 35).

**Figure 7:**
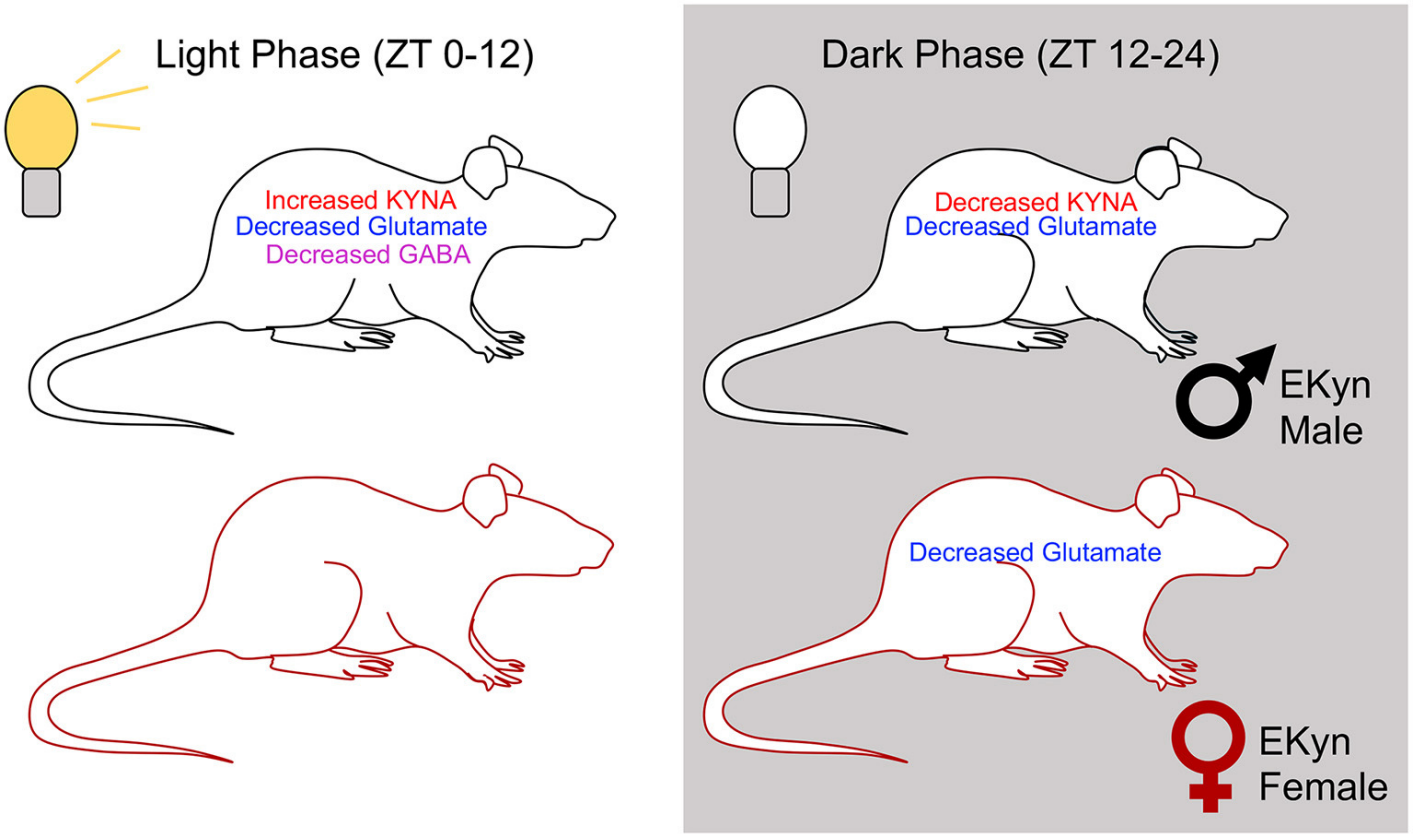
Summary figure representing overall trends in extracellular concentrations of KYNA, glutamate, and GABA in EKyn males and females across the two light phases.

In parallel to the observed diurnal fluctuations in glutamate, we determined a phase-dependent decrease in hippocampal GABA levels in EKyn male offspring compared to controls. These results are consistent with previous findings where acute local KYNA elevation dose-dependently decreases extracellular GABA levels in the brain (40). Yet curiously, in our EKyn paradigm, GABA levels are reduced in male offspring transiently, in a phase-dependent manner, after the late light phase elevation in KYNA levels. The temporal delay and alteration in extracellular GABA in the absence of elevated KYNA levels could potentially be explained by a transient disinhibition of α7nACh receptor activation on GABAergic interneurons from the stratum radiatum, which could create a GABA_A_ receptor-mediated negative feedback loop (51). Relating these present findings to the sleep and behavioral changes reported in male EKyn offspring, we speculate that reduced extracellular hippocampal GABA concentrations toward the end of the dark phase could be related to aberrant rapid eye movement (REM) sleep and contextual memory impairment observed in male EKyn offspring. REM sleep is tightly regulated by afferent medial septal GABAergic projections to the hippocampus, and when silenced, block the consolidation of contextual memory during REM sleep (37, 52). Interestingly, female EKyn offspring do not exhibit reduced GABA levels compared to their male EKyn counterparts, which may also be related to sex-specific changes in behavior and arousal previously reported (26, 27). However, sparse information exists on neurochemical profiles of female rats from studies using neurodevelopmental manipulations. Thereby, we presently provide novel information regarding hippocampal KYNA, GABA, and glutamate levels, while considering sex as a biological variable (See Figure 7).

As individuals with SZ and BD have elevated levels of KYNA in the brain (5, 11, 15), the enhanced KYNA found in the brain of adult EKyn rats presents critical translational value to investigate the longstanding ability of KYNA to influence multiple neuromodulatory systems implicated in the pathology of psychotic disorders. As described presently, several impairments observed in adult EKyn rats resemble hallmark neurochemical and behavioral deficits found in individuals with psychotic disorders including SZ and BD (53–57). EKyn rats exhibit neurochemical changes in hippocampal glutamate levels, analogous to reduced temporal lobe glutamate levels reported clinically (53, 55, 56). In patients with psychotic disorders, glutamatergic and GABAergic deficits have been linked to impairments in working and association memory, as well as increased risk for presentation of negative symptoms (58, 59).

Our findings also parallel neurochemical alterations observed in other prenatal insult paradigms that attempt to capture pathophysiological alterations common to psychotic disorders (30, 60–63). The contribution of each individual prenatal litter is an important consideration in studies like ours, and albeit a small sample size compared to clinical investigations, our results provide novel mechanistic insights regarding the neurodevelopmental implications for elevated KYNA and its impact on hippocampal excitatory and inhibitory neuromodulation. A misbalance of gating through excitation and inhibition is postulated to form the basis for cognitive and behavioral disturbances (64). Imbalances observed in GABA and glutamate levels may also be applicable to neurodevelopmental disorders such as autism spectrum disorders, where reduced GABA and glutamate levels are found in specific frontal, thalamic, and striatal brain regions (65, 66). Ultimately, the deficits in glutamatergic and GABAergic neuromodulation in relation to KYNA elevation in EKyn young adult offspring bridge our understanding between KYNA and neuromodulatory deficits which may contribute to the observed impairments in cognition, sleep, and arousal (21, 26, 27). In conclusion, sex-specific neurochemical changes observed in this study highlight the importance of evaluating sex as a biological variable when considering therapeutics strategies, including inhibition of KAT II to inhibit KYNA synthesis (48, 67, 68), and improve behavioral dysfunction and clinical outcomes for individuals suffering from psychiatric disorders.

## Data Availability Statement

The raw data supporting the conclusions of this article will be made available by the authors, without undue reservation.

## Ethics Statement

The animal study was reviewed and approved by Institutional Animal Care and Use Committees and were in accordance with the National Institutes of Health Guide for the Care and Use of Laboratory Animals at the University of South Carolina.

## Author Contributions

CW: conducted research, formal analysis, writing – original draft, writing – review & editing, visualization, and project administration. KR: conducted research, methodology, formal analysis, writing – original draft, writing – review & editing, and visualization. NW and AL: conducted research and writing – review & editing. SB: conceptualization, methodology, and writing – review & editing. AP: conceptualization, methodology, formal analysis, writing – original draft, writing – review & editing, visualization, supervision, project administration, and funding acquisition. All authors contributed to the article and approved the submitted version.

## Funding

This work was funded by the National Institutes of Health Grant Nos. NIH R01 NS102209, P50 MH103222, and support from the University of South Carolina (Magellan Scholar) and the University of South Carolina Honors College (Science Undergraduate Research Fellowships).

## Conflict of Interest

The authors declare that the research was conducted in the absence of any commercial or financial relationships that could be construed as a potential conflict of interest.

## Publisher's Note

All claims expressed in this article are solely those of the authors and do not necessarily represent those of their affiliated organizations, or those of the publisher, the editors and the reviewers. Any product that may be evaluated in this article, or claim that may be made by its manufacturer, is not guaranteed or endorsed by the publisher.

## Abbreviations

SZ: schizophrenia
BD: bipolar disorder
KYNA: kynurenic acid
EKyn: embryonic kynurenine treatment
ECon: embryonic control treatment
ZT: zeitgeber time
KP: kynurenine pathway
KYNA: kynurenic acid
NMDA: pertaining to the N-methyl-D-aspartate glutamate receptor
GABA: γ-aminobutyric acid
α7nACh: pertaining to the alpha 7 nicotinic acetylcholine receptor
ED: embryonic day
PD: postnatal day
AP: anterior-posterior
LM: lateral-medial
DV: dorsal-ventral
HPLC: high-performance liquid chromatography
UHPLC: ultra high-performance liquid chromatography
KAT II: kynurenine amino transferase II
REM: rapid eye movement.
