# Supplementary material for: Time of Day-Dependent Alterations in Hippocampal Kynurenic Acid, Glutamate, and GABA in Adult Rats Exposed to Elevated Kynurenic Acid During Neurodevelopment
Source: Front Psychiatry. 2021 Sep 17;12:734984. doi: 10.3389/fpsyt.2021.734984 (PMC8484637; doi:10.3389/fpsyt.2021.734984)
Supplement: Supplementary file 1 [file Data_Sheet_1.PDF]

Supplemental Figure 1: Visual representation of microdialysis probe tracks in the dorsal hippocampus. **(A)** ECon males. **(B)** EKyn males. **(C)** ECon females. **(D)** EKyn females.

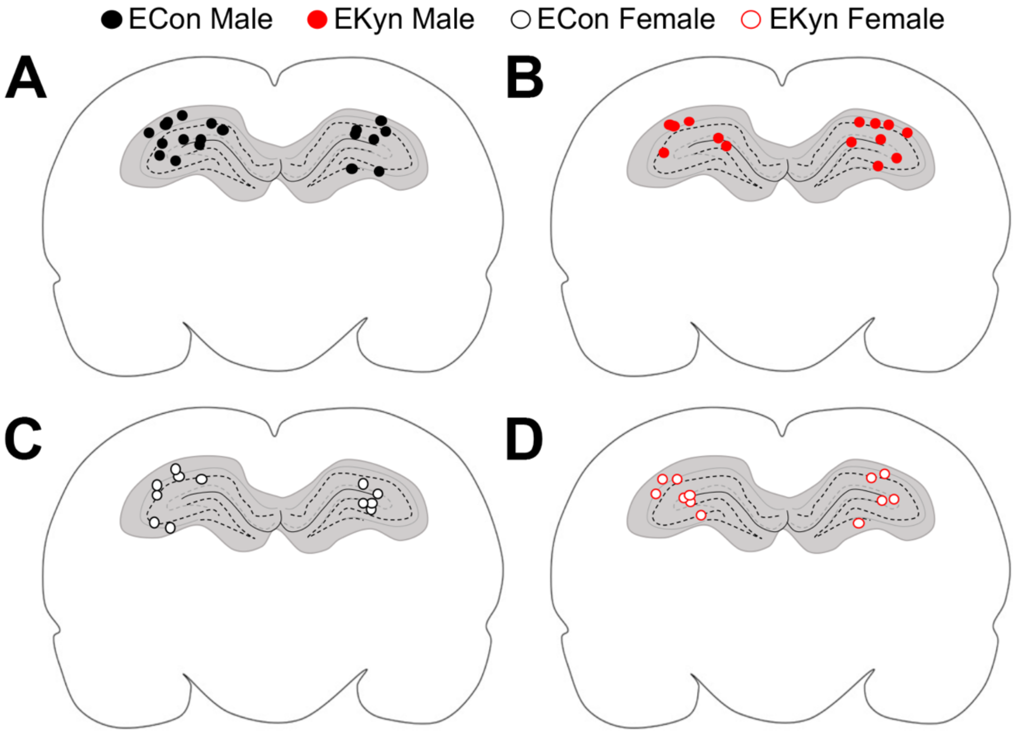

Figure	Data	Statistical Test	Interaction	F Statistic	P Value	Main Effect	F Statistic	P Value	Post Hoc Test
1C	Weight	3-Way RM ANOVA	PD x EKyn<br>PD x Sex<br>EKyn x Sex<br>PD x EKyn x Sex	F(3, 48) = 0.5302<br>F(3, 48) = 474.7<br>F(1, 48) = 1.185<br>F(3, 48) = 0.5650	P=0.6637<br>P<0.0001<br>P=0.2819<br>P=0.6407	PD<br>EKyn<br>Sex	F(3, 48) = 795.8 F(1,<br>48) = 0.2116 F(1, 48)<br>= 1906	P<0.0001<br>P=0.6476<br>P<0.0001	
		2-Way RM ANOVA	PD x EKyn	**M:** F(3, 34) = 0.51<br>**F:** F(3, 34) = 0.023	**M:** P=0.6731<br>**F:** P=0.9952	PD<br>EKyn	**M:** F(3, 34) = 2742<br>**F:** F(2.25, 25.55) = 3396<br>**M:** F(1, 14) = 0.64 **F:**<br>(1, 14) = 0.1888	**M:** P<0.0001<br>**F:** P<0.0001<br>**M:** P=0.4366<br>**F:** P=0.6705	
2A	Plasma Tryptophan	3-Way ANOVA	ZT x EKyn<br>ZT x Sex<br>EKyn x Sex<br>ZT x EKyn x Sex	F(1, 40) = 0.1828<br>F(1, 40) = 1.883<br>F(1, 40) = 0.00032<br>F(1, 40) = 0.6386	P=0.6713<br>P=0.1776<br>P=0.9858<br>P=0.4289	ZT<br>EKyn<br>Sex	F(1, 40) = 0.04681<br>F(1, 40) = 0.4654<br>F(1, 40) = 7.658	P=0.8298<br>P=0.4990<br>P=0.0085	
		2-Way ANOVA	ZT x EKyn	**M:** F(1, 27) = 0.1709<br>**F:** F(1, 13) = 0.3084	**M:** P=0.6826<br>**F:** P=0.5881	ZT<br>EKyn	**M:** F(1, 27) = 3.124<br>**F:** F(1, 13) = 0.2739<br>**M:** F(1, 27) = 0.5463<br>**F:** F(1, 13) = 0.1005	**M:** P=0.0885<br>**F:** P=0.6095<br>**M:** P=0.4662<br>**F:** P=0.7563	
2B	Plasma Kynurenine	3-Way ANOVA	ZT x EKyn<br>ZT x Sex<br>EKyn x Sex<br>ZT x EKyn x Sex	F(1, 41) = 0.2657<br>F(1, 41) = 1.337<br>F(1, 41) = 0.8584<br>F(1, 41) = 0.6011	P=0.6090<br>P=0.2543<br>P=0.3596<br>P=0.4426	ZT<br>EKyn<br>Sex	F(1, 41) = 2.970<br>F(1, 41) = 0.2720<br>F(1, 41) = 7.640	P=0.0923<br>P=0.6048<br>P=0.0085	
		2-Way ANOVA	ZT x EKyn	**M:** F(1, 27) = 0.0486<br>**F:** F(1, 14) = 0.6114	**M:** P=0.8271<br>**F:** P= 0.4473	ZT<br>EKyn	**M:** F(1, 27) = 0.2319<br>**F:** F(1, 14) = 3.043<br>**M:** F(1, 27) = 0.1181<br>**F:** F(1, 14) = 0.7695	**M:** P=0.6340<br>**F:** P=0.1030<br>**M:** P=0.7337<br>**F:** P=0.3952	
2C	Plasma KYNA	3-Way ANOVA	ZT x EKyn<br>ZT x Sex<br>EKyn x Sex<br>ZT x EKyn x Sex	F(1, 41) = 0.008477<br>F(1, 41) = 0.5215<br>F(1, 41) = 0.2151<br>F(1, 41) = 0.2092	P=0.9271<br>P=0.4743<br>P=0.6452<br>P=0.6498	ZT<br>EKyn<br>Sex	F(1, 41) = 2.071<br>F(1, 41) = 0.022111<br>F(1, 41) = 11.53	P=0.1577<br>P=0.8825<br>P=0.0015	
		2-Way ANOVA	ZT x EKyn	**M:** F(1, 27) = 0.1148<br>**F:** F(1, 14) = 0.08620	**M:** P=0.7374<br>**F:** P=0.7734	ZT<br>EKyn	**M:** F(1, 27) = 0.4420<br>**F:** F(1, 14) = 1.334<br>**M:** F(1, 27) = 0.08539<br>**F:** F(1, 14) = 0.1071	**M:** P=0.5118<br>**F:** P=0.2675<br>**M:** P=0.7724<br>**F:** P=0.7483	
3	Hippocampal KYNA	3-Way ANOVA	ZT x EKyn<br>ZT x Sex<br>EKyn x Sex<br>ZT x EKyn x Sex	F(3, 107) = 0.4272<br>F(3, 107) = 0.5229<br>F(1, 107) = 0.5491<br>F(3, 107) = 0.6343	P=0.7339<br>P=0.6674<br>P=0.4603<br>P=0.5945	ZT<br>EKyn<br>Sex	F(3, 107) = 1.054<br>F(1, 107) = 4.879<br>F(1, 107) = 0.8291	P=0.3719<br>P=0.0293<br>P=0.3646	
		2-Way ANOVA	ZT x EKyn	**M:** F(3, 58) = 0.8708<br>**F:** F(3, 49) = 0.2044	**M:** P=0.4615<br>**F:** P=0.8929	ZT<br>EKyn	**M:** F(3, 58) = 0.5965<br>**F:** F(3, 49) = 1.396<br>**M:** F(1, 58) = 3.879<br>**F:** F(1, 49) = 1.442	**M:** P=0.6198<br>**F:** P=0.2552<br>**M:** P=0.0537<br>**F:** P=0.2356	**M:** ECon vs EKyn (ZT 6), P=0.0500
4A	Extracellular KYNA Light Phase	3-Way ANOVA	ZT x EKyn<br>ZT x Sex<br>EKyn x Sex<br>ZT x EKyn x Sex	F(11, 207) = 0.8705<br>F(11, 207) = 0.5496<br>F(1, 207) = 10.01<br>F(11, 207) = 0.3905	P=0.5701<br>P=0.8675<br>P=0.0018<br>P=0.9588	ZT<br>EKyn<br>Sex	F(11, 207) = 0.5886<br>F(1, 207) = 10.62<br>F(1, 207) = 2.913	P=0.8371<br>P=0.0013<br>P=0.0894	
		2-Way ANOVA	ZT x EKyn	**M:** F(11, 87) = 0.6576<br>**F:** F(11, 120) = 0.3615	**M:** P=0.7742<br>**F:** P=0.9682	ZT<br>EKyn	**M:** F(11, 87) = 0.6968<br>**F:** F(11, 120) = 0.1352<br>**M:** F(1, 87) = 13.39<br>**F:** F(1, 120) = 0.0072	**M:** P=0.7382<br>**F:** P=0.9996<br>**M:** P=0.0004<br>**F:** P=0.9326	**M:** ECon vs EKyn (ZT 8), P=0.0282<br>**M:** ECon vs EKyn (ZT 9), P=0.0312<br>**M:** ECon vs EKyn (ZT 10), P=0.0367
4B	Extracellular KYNA Dark Phase	3-Way ANOVA	ZT x EKyn<br>ZT x Sex<br>EKyn x Sex<br>ZT x EKyn x Sex	F(11, 160) = 0.3549<br>F(11, 160) = 0.3534<br>F(1, 160) = 6.744<br>F(11, 160) = 0.3205	P=0.9709<br>P=0.9714<br>P=0.0103<br>P=0.9805	ZT<br>EKyn<br>Sex	F(11, 160) = 0.8451<br>F(1, 160) = 0.7263<br>F(1, 160) = 6.635	P=0.5954<br>P=0.3954<br>P=0.0109	

		2-Way ANOVA	ZT x EKyn	**M:** F(11, 68) = 0.2609<br>**F:** F(11, 92) = 0.4161	**M:** P=0.9908<br>**F:** P=0.9456	ZT<br>EKyn	**M:** F(11, 68) = 0.7105<br>**F:** F(11, 92) = 0.3429<br>**M:** F(1, 68) = 4.556<br>**F:** F(1, 92) = 1.955	**M:** P=0.7244<br>**F:** P=0.9734<br>**M:** P=0.0364<br>**F:** P=0.1654	
	Extracellular KYNA 6-hr Summary	3-Way ANOVA	ZT x EKyn<br>ZT x Sex<br>EKyn x Sex<br>ZT x EKyn x Sex	F(3, 46) = 6.364<br>F(3, 46) = 0.7105<br>F(1, 24) = 0.02618<br>F(3, 46) = 5.242	P=0.0011<br>P=0.5507<br>P=0.8728<br>P=0.0034	ZT<br>EKyn<br>Sex	F(3, 46) = 1.563<br>F(1, 24) = 0.004321<br>F(1, 24) = 1.580	P=0.211<br>P=0.9481<br>P=0.2209	
		2-Way ANOVA	ZT x EKyn	**M:** F(3, 19) = 5.279<br>**F:** F(3, 27) = 1.200	**M:** P=0.0081<br>**F:** P=0.3287	ZT<br>EKyn	**M:** F(3, 19) = 0.8473<br>**F:** F(3, 27) = 1.723<br>**M:** F(1, 11) = 0.0001<br>**F:** F(1, 13) = 0.0006	**M:** P=0.4851<br>**F:** P=0.1858<br>**M:** P=0.9916<br>**F:** P=0.9799	**M:** ECon vs EKyn (ZT 6-12), P=0.0282<br>**M:** ZT 0-6 vs ZT 18-24 (ECon), P=0.0440<br>**M:** ZT 6-12 vs ZT 12-18 (EKyn), P=0.0091<br>**M:** ZT 6-12 vs ZT 18-24 (EKyn), P=0.0038
5A	Extracellular Glutamate Light Phase	3-Way ANOVA	ZT x EKyn<br>ZT x Sex<br>EKyn x Sex<br>ZT x EKyn x Sex	F(11, 311) = 0.4099<br>F(11, 311) = 0.6066<br>F(1, 311) = 3.588<br>F(11, 311) = 0.3305	P=0.9513<br>P=0.8231<br>P=0.0602<br>P=0.9787	ZT<br>EKyn<br>Sex	F(11, 311) = 0.9211<br>F(1, 311) = 6.984<br>F(1, 311) = 1.987	P=0.5202<br>P=0.0086<br>P=0.1597	
		2-Way ANOVA	ZT x EKyn	**M:** F(11, 113) = 0.2894<br>**F:** F(11, 198) = 0.5160	**M:** P=0.9868<br>**F:** P=0.8913	ZT<br>Ekyn	**M:** F(11, 113) = 1.064<br>**F:** F(11, 198) = 0.3669<br>**M:** F(1, 113) = 8.616<br>**F:** F(1, 198) = 0.3731	**M:** P=0.3968<br>**F:** P=0.9673<br>**M:** P=0.0040<br>**F:** P=0.5420	**M:** ECon vs EKyn (ZT 9), P=0.0457
5B	Extracellular Glutamate Dark Phase	3-Way ANOVA	ZT x EKyn<br>ZT x Sex<br>EKyn x Sex<br>ZT x EKyn x Sex	F(11, 179) = 1.743<br>F(11, 179) = 1.065<br>F(1, 179) = 0.03350<br>F(11, 179) = 0.3890	P=0.0673<br>P=0.3922<br>P=0.8550<br>P=0.9591	ZT<br>EKyn<br>Sex	F(11, 179) = 2.941<br>F(1, 179) = 22.40<br>F(1, 179) = 6.416	P=0.0013<br>P<0.0001<br>P=0.0122	
		2-Way ANOVA	ZT x EKyn	**M:** F(11, 71) = 1.146<br>**F:** F(11, 108) = 1.061	**M:** P=0.3399<br>**F:** P=0.3992	ZT<br>EKyn	**M:** F(11, 71) = 2.917<br>**F:** F(11, 108) = 1.006<br>**M:** F(1, 71) = 9.772<br>**F:** F(1, 108) = 13.77	**M:** P=0.0032<br>**F:** P=0.4463<br>**M:** P=0.0026<br>**F:** P=0.0003	**M:** ECon vs EKyn (ZT 13), P=0.0169<br>**M:** ECon vs EKyn (ZT 14), P=0.0064<br>**F:** ECon vs EKyn (ZT 13), P=0.0036<br>**F:** ECon vs EKyn (ZT 14), P=0.0027
5C	Extracellular Glutamate 6hr Summary	3-Way ANOVA	ZT x EKyn<br>ZT x Sex<br>EKyn x Sex<br>ZT x EKyn x Sex	F(3, 68) = 0.7430<br>F(3, 68) = 0.3590<br>F(1, 35) = 0.8913<br>F(3, 68) = 0.2216	P=0.5301<br>P=0.7828<br>P=0.3516<br>P=0.8811	ZT<br>EKyn<br>Sex	F(3, 68) = 4.034<br>F(1, 35) = 4.040<br>F(1, 35) = 1.803	P=0.0106<br>P=0.0522<br>P=0.1880	
		2-Way ANOVA	ZT x EKyn	**M:** F(3, 26) = 0.3471<br>**F:** F(3, 42) = 0.7995	**M:** P=0.7915<br>**F:** P=0.5011	ZT<br>EKyn	**M:** F(3, 26) = 4.056<br>**F:** F(3, 42) = 1.640<br>**M:** F(1, 15) = 2.726<br>**F:** F(1, 20) = 0.8212	**M:** P=0.0172<br>**F:** P=0.1945<br>**M:** P=0.1195<br>**F:** P=0.3756	**M:** ZT 0-6 vs ZT 18-24 (ECon), P=0.0453<br>**M:** ZT 0-6 vs ZT 6-12 (EKyn), P= 0.0303<br>**M:** ZT 0-6 vs ZT 12-18 (EKyn), P=0.0318<br>**M:** ZT 0-6 vs ZT 18-24 (EKyn), P=0.0258
6A	Extracellular GABA Light Phase	3-Way ANOVA	ZT x EKyn<br>ZT x Sex<br>EKyn x Sex<br>ZT x EKyn x Sex	F(11, 256) = 0.3486<br>F(11, 256) = 1.335<br>F(1, 256) = 0.1467<br>F(11, 256) = 0.6781	P=0.9735<br>P=0.2053<br>P=0.7020<br>P=0.7589	ZT<br>EKyn<br>Sex	F(11, 256) = 1.793<br>F(1, 256) = 0.4611<br>F(1, 256) = 32.54	P=0.0554<br>P=0.4977<br>P<0.0001	
		2-Way ANOVA	ZT x EKyn	**M:** F(11, 113) = 0.3415<br>**F:** F(11, 143) = 0.8661	**M:** P=0.9743<br>**F:** P=0.5750	ZT<br>EKyn	**M:** F(11, 113) = 1.673<br>**F:** F(11, 143) = 0.4471<br>**M:** F(1, 113) = 0.3286<br>**F:** F(1, 143) = 0.08773	**M:** P=0.0886<br>**F:** P=0.9318<br>**M:** P=0.5676<br>**F:** P=0.7675	
6B	Extracellular GABA Dark Phase	3-Way ANOVA	ZT x EKyn<br>ZT x Sex<br>EKyn x Sex<br>ZT x EKyn x Sex	F(11, 166) = 0.5605<br>F(11, 166) = 0.5835<br>F(1, 166) = 9.017<br>F(11, 166) = 0.2196	P=0.8586<br>P=0.8405<br>P=0.0031<br>P=0.9961	ZT<br>EKyn<br>Sex	F(11, 166) = 0.9988<br>F(1, 166) = 7.170<br>F(1, 166) = 4.213	P=0.9988<br>P=0.0082<br>P=0.0417	
		2-Way ANOVA	ZT x EKyn	**M:** F(11, 76) = 0.4368<br>**F:** F(11, 90) = 0.4071	**M:** P=0.9345<br>**F:** P=0.9496	ZT<br>EKyn	**M:** F(11, 76) = 0.5426<br>**F:** F(11, 90) = 0.3421<br>**M:** F(1, 76) = 23.00<br>**F:** F(1, 90) = 0.04528	**M:** P=0.8680<br>**F:** P=0.9736<br>**M:** P<0.0001<br>**F:** P=0.8320	**M:** ECon vs EKyn (ZT 14), P=0.0312<br>**M:** ECon vs EKyn (ZT 16), P=0.0075<br>**M:** ECon vs EKyn (ZT 21), P=0.0430

6C	Extracellular GABA 6hr Summary	3-Way ANOVA	ZT x EKyn<br>ZT x Sex<br>EKyn x Sex<br>ZT x EKyn x Sex	F(3, 62) = 0.4237<br>F(3, 62) = 1.516<br>F(1, 31) = 1.588<br>F(3, 62) = 1.184	P=0.7367<br>P=0.2192<br>P=0.2170<br>P=0.3232	ZT<br>EKyn<br>Sex	F(3, 62) = 2.727<br>F(1, 31) = 0.3916<br>F(1, 31) = 6.548	P=0.0516<br>P=0.5360<br>P=0.0156	
		2-Way ANOVA	ZT x EKyn	**M:** F(3, 27) = 1.020<br>**F:** F(3, 35) = 0.3813	**M:** P=0.3994<br>**F:** P=0.7671	ZT<br>EKyn	**M:** F(3, 27) = 2.462<br>**F:** F(3, 35) = 1.127<br>**M:** F(1, 14) = 1.499<br>**F:** F(1, 17) = 0.3813	**M:** P=0.0841<br>**F:** P=0.3516<br>**M:** P=0.2410<br>**F:** P=0.6217	

Supplemental Table 1: Summary of statistical analysis. List of abbreviations: postnatal day (PD), embryonic kynurenine (EKyn), embryonic control (ECon), Zeitgeber time (ZT), kynurenic acid (KYNA), γ-amino-butyric acid (GABA), male (M), female (F).

	Males		Females	
**Weight**	**ECon**	**EKyn**	**ECon**	**EKyn**
PD 21	8	8	8	8
PD 35	8	8	8	8
PD 47	4	4	4	4
PD 56	8	8	8	8

Supplemental Table 2: Number of samples in weight analyses. List of abbreviations: embryonic control treatment (ECon), embryonic kynurenine treatment (EKyn), postnatal day (PD).

	Males		Females	
**Plasma Tryptophan**	**ECon**	**EKyn**	**ECon**	**EKyn**
ZT 0	7	7	5	5
ZT 12	8	8	4	3
**Plasma Kynurenine**				
ZT 0	7	7	5	5
ZT 12	8	9	5	3
**Plasma KYNA**				
ZT 0	7	7	5	5
ZT 12	8	9	5	3
**Hippocampal KYNA**				
ZT 0	7	7	5	5
ZT 6	10	11	12	10
ZT 12	8	7	5	4
ZT 18	8	8	8	8

Supplemental Table 3: Number of samples in biochemical plasma and tissue analyses**.** List of abbreviations: embryonic control treatment (ECon), embryonic kynurenine treatment (EKyn), Zeitgeber time (ZT), kynurenic acid (KYNA).

	Males		Females	
**Extracellular Metabolites**	**ECon**	**EKyn**	**ECon**	**EKyn**
KYNA	6	6	6	7
	4	4	6	5
Glutamate	8	7	10	12
	5	5	6	7
GABA	8	7	8	11
	6	4	5	6

Supplemental Table 4: Number of samples in microdialysis extracellular analyses. Light phase is denoted by white background and dark phase is denoted by gray background in table. List of abbreviations: embryonic control treatment (ECon), embryonic kynurenine treatment (EKyn), kynurenic acid (KYNA), y-aminobutyric acid (GABA).
